# *In vitro *culture of *Plasmodium berghei*-ANKA maintains infectivity of mouse erythrocytes inducing cerebral malaria

**DOI:** 10.1186/1475-2875-10-346

**Published:** 2011-11-25

**Authors:** Ronan Jambou, Fatima El-Assaad, Valery Combes, Georges E Grau

**Affiliations:** 1 Vascular Immunology Unit, Department of Pathology and Bosch Institute, Sydney Medical School, The University of Sydney, NSW, Australia; 2 Institut Pasteur de Madagascar, BP1274 Antananarivo 101, Antananarivo, Madagascar

## Abstract

**Background:**

Infection with *Plasmodium berghei *is a widely used model of murine malaria and a powerful tool for reverse genetic and pathogenesis studies. However, the efficacy of *in vitro *reinvasion of erythrocytes is generally low, limiting *in vitro *studies.

**Methods:**

*Plasmodium berghei *ANKA-infected blood obtained from a susceptible infected mouse was cultured in various conditions and *in vitro *parasitaemia was measured every day to evaluate the rate of reinvasion.

**Results:**

High quality culture media were used and reinvasion rates were improved by vigorous orbital shaking of the flask and increasing density of the medium with gelatin.

**Discussion:**

Using these settings, reinvasion of normal mouse erythrocytes by the parasite was obtained *in vitro *over two weeks with preservation of the infectivity *in vivo*.

## Background

*Plasmodium berghei *is an African murine malaria parasite isolated by Vincke and Bafort in Katanga (PbNK for New York-Katanga) and in Kasapa (PbANKA for Antwerpen-Kasapa) [1,2]. During natural infection, the blood stages of the parasite undergo asynchronous development with a haploid cycle of 22 hours. Their preference for immature red blood cells (RBC) [1,3,4] suggests that they belong to the "vivax group". The gametocyte production is intense, with 20% of merozoites of each asexual cycle developing into gametocytes within 24 hours in phenylhydrazine-treated mice. Despite the phylogenetic distance, the conservation of housekeeping genes and of biochemical as well as genetic processes between human and murine parasites provided the first justification for their use in malaria research. Infection of mice with *Plasmodium berghei *and *Plasmodium yoelii *established the first murine models of malaria, with early studies utilizing these models to test drug efficacy (the *Peter's model*).

The first attempt to maintain the parasite *in vitro *was performed by serial passages through rats and tissue culture. These passages were associated with a sharp decrease in the virulence of the parasite *in vivo *[5]. Early studies developed culture conditions, which permitted the combination of *in vivo *and *in vitro *experiments with the same parasites [4-6], and methods for *in vitro *culture of gametocytes were rapidly described. Liver stages of *P. berghei *were obtained in the HepG2 cell line, which made it possible to study prophylactic drug efficacy [7]. *In vitro *development of *Plasmodium *ookinetes was achieved later on, followed by a complete *in vitro *development of mosquito phases. Several strains with the same genetic background [8] have been derived from the original isolates with different sensitivity to drugs. They are used for drug testing in maturation assays, molecular studies or evaluation of vaccine efficacy. Selection of mouse strains with various degrees of sensitivity for the parasite paved the way for analysis of the pathophysiology of cerebral malaria (CM) [9,10] and of severe anemia [11].

A new area of interest opened up with the establishment of protocols to knock-out specific genes in the parasite to investigate gene function. However, these reverse genetic studies (reviewed by [12]) are still limited by i) a weak transfection efficiency between 10^-3 ^to 10^-4 ^whereas efficiency reach 10^-2 ^in *Toxoplasma gondii)*, ii) the limited number of markers available to counter select transfectants iii) the mechanism of integration of foreign DNA (nearly always through homologous recombination), and iv) by the time required to select transfected parasites. Indeed the pyrimethamine treatment required to select transfectants is usually performed only 10 to 15 days after injection of the parasite into mice. Using these methods GFP fluorescent parasites were obtained, which created new areas of application. Maintaining parasites *in vitro *for a long period of time would allow selection of transfectants *in vitro*. This could pave the way for *in vitro *studies and facilitate infection of mice by these parasites by reducing competition between wild and mutated parasites in the mouse.

## Methods

### Maintenance of *Plasmodium berghei*

The *P. berghei *ANKA (PbA) strain was obtained from Josef Bafort and maintained [9] by successive infection of CBA/Ca and C57BL/6 mice. Each mouse was inoculated with 10^6 ^IRBC intra-peritoneally [10]. For each culture condition, mice were bled 7 days post-infection (when parasitaemia in blood reached more than 8%) and infected red blood cells (IRBC) were cultured after careful removal of leukocytes. Freezing of the IRBC in liquid nitrogen was done in Alsever's solution, as described [10]. All experiments complied with the Australian guidelines for animal research and protocol # K20/7-2006/3/4434 was approved by the University of Sydney Animal Ethics Committee.

### Maintenance of in vitro culture

Culture medium was removed daily after centrifugation of the culture at 600 g for 10 min. The pellet was diluted at 2.5% haematocrit with fresh medium. Non-infected red blood cells (NRBC) were obtained from healthy mice, and were added twice a week after platelets had been carefully removed from the blood by two washes and centrifugation at 400 g. Buffy coat was removed following Janse *et al *[13]. Culture was conducted in 75 cm^2 ^flasks, flushed with gas (5% CO_2_, 5% O_2_, 90% N_2_) and totally sealed. Effect of storage at 4°C of NRBC and of IRBC was checked by keeping cells in the fridge during increasing time before seeding. Selection of mature stages of the parasite was performed on an AutoMacs (Miltenyi Biotec) using the "sensitive" procedure with IRBC diluted 1:20 in PBS. Duration of the parasite life cycle and identification of the stage of the parasite in culture were evaluated by repeated Giemsa-stained thin smears on sampled IRBC.

### Stability of the mouse RBC in culture

Starting culture conditions followed those described by Mons *et al *and Janse *et al *[6,13]. To avoid mild haemolysis of IRBC in culture, tests were conducted to adapt the density of the medium and the method of shaking. Concentrations of fetal calf serum (FCS) from 5 to 20% and gelatin from 0.5 to 5% were tested. IRBC were added to RPMI1640 medium supplemented by these components and maintained at 37°C for five days. Medium was changed daily and haemolysis was checked optically at each step. In parallel, several ways of agitating the culture were tried, including magnetic stirring and permanent orbital shaking to minimize haemolysis.

### Reinvasion study

Rate of reinvasion was calculated by daily determination of the parasitaemia in culture, using thin Giemsa-stained smears. Parasites were counted on 100 fields at ×1, 000 magnification. Reinvasion of newly added red blood cells by the parasites after several days of culture was experimentally confirmed by labeling of i) NRBC with a green fluorescent dye (PKH-67, MINI67-IKT, Sigma) prior to addition to the culture flask and ii) parasites with hydroethidine (red fluorescence). Parasites were labeled by incubation with hydroethidine for 30 min at 37°C, two days after the addition of green-NRBC to the culture. Double-labeled IRBC were observed with a confocal microscope (Olympus FV1000).

### Infectivity experiment

In vivo infectivity of the cultured parasites was evaluated by inoculating IRBC in susceptible mice (CBA/Ca or C57BL/6). After 12 days of culture 10^6 ^IRBCs were injected intra-peritoneally [10] and parasitaemia was monitored daily on thin tail vein blood smears from day 5 post-infection onwards..

### Final composition of the medium

The final composition of the medium was: RPMI1640 (3/4): DMEM-F12 (1/4); bicarbonate 32 mM, HEPES 25 mM; Albumax II 0.5%; glucose 3 g/L; hypoxanthine 200 μM; calcium 2 mM; gelatin 0.1%; choline 1 mM. Chemicals and dyes were purchased from Sigma, except for AlbuMAX II (Gibco n°10021-037), DMEM/F12 (with L-glutamine, Invitrogen n°11330), hydroethidine (Polysciences n°17084) and culture flasks (75 cm^2^, Corning). Cultures were maintained at 32°C and medium was changed daily. Fresh mouse NRBCs were added twice a week (dilution 1:4). Haematocrit was maintained at 2.5%. Culture flasks were maintained closed and vertical, filled with no more than 30 mL and gased with a 5% CO_2_, 5% O_2_, 90% N_2 _gas mixture. Permanent shaking at 100 rpm on an orbital shaker was applied.

## Results

### Stability of red blood cells

**Figure 1:**
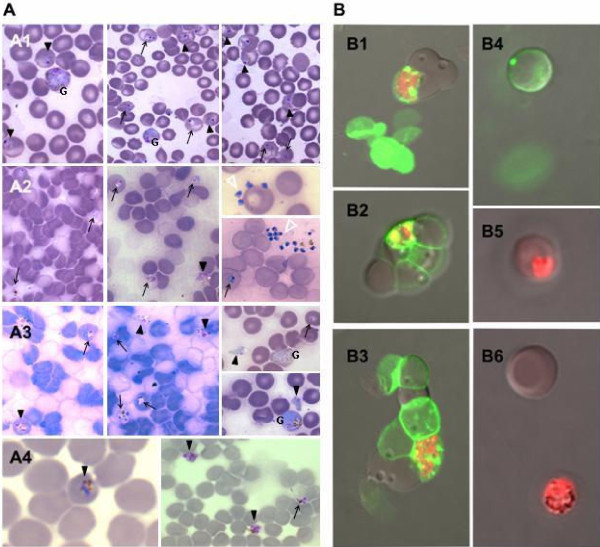
**Aspect of *in vitro *culture of *P. berghei *in mouse erythrocytes**. A/Follow up of the cultures. Schizonts burst and reinvasion is followed up on Giemsa-stained thin smears. Late stage (arrow head), early stage (arrow), merozoites (empty arrow head), gametocytes (G) A1: aspects of culture at day 3 after seeding showing early and late stages and gametocytes; A2: aspects of culture at day 4 with early and late stages and release of merozoites; A3: aspects of culture at day 5 showing early and late stages and release of merozoites; A4: aspects of culture at day 7 showing trophozoites. B/Double fluorescent labelling of erythrocytes and parasites To confirm reinvasion of red blood cells (RBC) by merozoites, RBC are labeled with a green fluorescent dye (PKH-67) prior to addition to the culture flask. They are added to the parasite culture at a ratio of 1:4. After two days of culture, parasites are labeled using hydroethidine (red fluorescent dye). Double-labeled IRBC are observed with a confocal microscope (Olympus FV1000, ×60). B1-3: aspect of IRBC after co-culture with green labelled RBC (bright field, red and green fluorescence merged). B4: green labelled RBC before adjunction to culture; B5-6: hydroethidine labelled parasites in culture (before adjunction of green RBC).

The stability of mouse RBC in culture was the first parameter that needed improving. As previously described, mouse IRBC maintained in culture at 37°C [14] presented mild haemolysis. During this study addition of 20% FCS to the medium significantly reduced this haemolysis, but consequently decreased maturation rate of the parasites after several days of culture. Haemolysis was totally abolished by addition of gelatin. Along the same line, permanent orbital shaking of the flask at 100 rpm achieved substantial mechanical disruption of the late schizonts (Figure 1) without inducing red cell alterations as seen with the magnetic stirrer. Using this setting, large numbers of well-separated merozoites were observed in culture (Figure 1A2) and the position of the merozoites on the RBC as well as merozoites inside RBC was readily detected (Figure 1A2).

### Merozoite production and reinvasion

In RPMI1640 medium supplemented in the same way as for *Plasmodium falciparum *[15], a decrease in the number of merozoites produced by parasites, i.e. the number of nuclei in schizonts, was observed after three life cycles of the parasite. This suggested insufficiency in nutrient levels, which was not overcome by increasing FCS concentration to more than 5% [13]. Medium composition was modified by addition of DMEM/F12 to RPMI1640. This medium is also compatible with endothelial cell culture. Based on the follow up of *in vitro *parasitaemia, numerous experiments were conducted, allowing optimization of the culture medium mixture to one part DMEM/F12 and three parts RPMI1640. Higher proportion of DMEM/F12 induced a reduction of the parasitaemia. Similarly, ionic concentration in the medium was adapted in agreement with the mouse serum concentrations [16]. Addition of calcium improved their maintenance in culture. Due to a high rate of parasite multiplication and acidification of the medium, the bicarbonate concentration was increased and culture media was changed daily. The temperature was also decreased to 32°C without apparent change in the duration of the life cycle of the parasite. Reinvasion of newly added red blood cell by the parasites was confirmed by dual labeling of NRBC and parasites with hydroethidine (Figure 1B).

**Figure 2:**
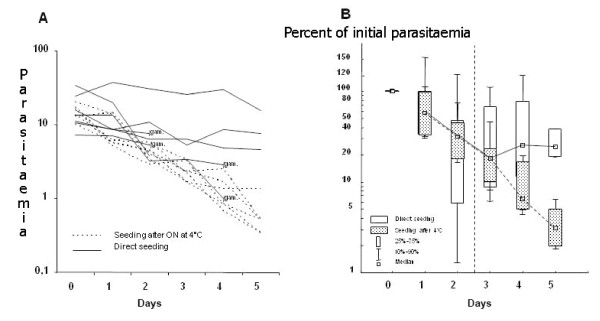
***In vitro *reinvasion rate of mouse erythrocytes by *P. berghei***. A/Effect of the storage of *P. berghei *ANKA before seeding. Isolates from 6 mice are seeded immediately after bleeding (direct seeding) and 6 after overnight storage at 4°C. Depending on the mouse, some isolates turned rapidly to gametocyte production (gam). No adjunction of RBC was done in the cultures. B/Follow up of parasitaemia after seeding of 18 separate isolates Isolates from 9 mice are seeded immediately after bleeding (direct seeding) and 9 after overnight storage of the infected blood at 4°C (ON). RBC are added after 2 days of culture, as marked by the dotted line (dilution 1:1 RBC:IRBC). After five days of culture most of the isolates seeded immediately after bleeding still harboured more than 30% of their initial parasitaemia (at the seeding day). After 12 days of culture parasitaemia of the isolates (direct seeding) are usually between 1 to 5% (data not shown). This is a different set of mice from Figure 2A.

After seeding in culture, parasitaemia could be maintained between 5 to 15% for 5 to 7 days (Figure 2) with fresh blood being added twice a week. After 12 days most of the cultures had still 0.5 to 1% parasitaemia despite a dilution of the IRBC 1:4 with NRBC twice a week. Storage of the normal RBC at 4°C for up to one week before use in culture had no effect on *in vitro *invasion. In contrast, storage of the IRBC at 4°C, either before seeding or after a couple of days of culture, had a dramatic effect on reinvasion (Figure 2) as already described for merozoites [17]. In most flasks, gametocyte production was rapidly observed and associated with a sharp decrease of the asexual parasitaemia (Figure 2A).

Selection of mature stage of the parasite, from infected blood was easily obtained using the AutoMacs in the same conditions as those used for *P. falciparum*. Following separation, parasitaemia could reach up to 90%.

### Infectivity

Infectivity of the cultured parasites for the host was assessed by re-injecting IRBC in mice either directly from cultures or after freezing cultured parasites in liquid nitrogen. Repeated cycles of *in vitro *culture of PbA did not affect the infectivity of the parasite for mice. After 12 days of culture, injection of 10^6 ^IRBC in susceptible CBA/J mice readily induced 2% to 6% of parasitaemia within 7 days. Mice with high parasitaemia displayed clear signs of CM.

## Discussion

In most protocols, early culture conditions for *P. berghei *closely resembled those of *P. falciparum *[13], as the maturation of young blood stages into schizonts is straightforward [18]. Long-term *in vitro *cultivation of *P. berghei *was claimed to be obtained by Ramaiya *et al *[18] at 27°C, and by Smalley [14] who achieved a low multiplication rate at 15°C. However, at 37°C, a decrease in parasite density was always observed as a consequence of the instability of the red blood cells (RBC). During all these studies, re-invasion appeared to be the bottleneck of the long-term cultivation of *P. berghei*. This was attributed to two main points: i) a restricted ability to invade mature RBC, which required adding blood with a high percentage of reticulocytes to the cultures, and ii) release of merozoites staying packed together or surrounded by RBC membrane preventing them from invading new cells. Schizonts can indeed survive for more than 20 hours in culture and most of the authors used a magnetic stirrer to increase the disruption of the IRBC. During this study, conditions of culture were adjusted to allow an average of 10% *in vitro *parasitaemia during the first week after seeding and at least 0.5 to 1% after 10 days. Mature stages of the parasite were easily purified using the AutoMacs procedure.

Preference of *P. berghei *for mouse reticulocytes has been widely reported and in "CM resistant" mice parasitaemia can reach 80% of RBC [10,11] supposed to be mostly reticulocytes. This has implications for *in vitro *studies of drug sensitivity or cell/cell interactions. In this study, mice used to provide NRBC for culture were not treated before bleeding to increase reticulocytosis. Nevertheless their blood allowed regular reinvasion of RBC by parasites. This protocol for culture of PbA-IRBC *in vitro *provides significant advantages particularly in allowing *in vitro *studies of co-culture with endothelial cells or leukocytes in conditions similar to human malaria. Cultivation of the parasites in this setting does not induce any loss of infectivity for mice.

## Competing interests

The authors declare that they have no competing interests.

## Authors' contributions

RJ designed the study and performed all the adaptation of the *in vitro *system and the confocal analysis; FEA and VC performed the *in vivo *studies; GEG provided support for the study, the scientific background on the *Plasmodium berghei *ANKA model and helped in the writing of the paper. All authors read and approved the final manuscript.
